# The Impact of Fissure-Adjacent Localization on Rupture Risk in Pulmonary Hydatid Disease

**DOI:** 10.7759/cureus.87899

**Published:** 2025-07-14

**Authors:** Suleyman Emre Akin, Furkan Cagri Oguzlar, Veysel Atilla Ayyildiz, Hasan Ekrem Camas, Isa Dongel, Rasih Yazkan

**Affiliations:** 1 Thoracic Surgery Department, Suleyman Demirel University Faculty of Medicine, Isparta, TUR; 2 Emergency Medicine Department, Suleyman Demirel University Faculty of Medicine, Isparta, TUR; 3 Radiology Department, Kocaeli City Hospital, Kocaeli, TUR

**Keywords:** cyst rupture, echinococcosis, fissure relationship, pulmonary hydatid cyst, risk factors, thoracic surgery

## Abstract

Background

Pulmonary hydatid cyst (PHC) rupture is a serious complication that can increase surgical difficulty and postoperative morbidity. Identifying preoperative predictors of rupture is essential for patient risk stratification and optimal surgical timing. This study aimed to evaluate clinical, radiological, and laboratory factors associated with cyst rupture, with a particular focus on the anatomical relationship with lung fissures.

Methods

A retrospective analysis was conducted on 37 patients who underwent surgery for pulmonary hydatid cysts between January 2012 and December 2021. Patients were categorized into ruptured and intact cyst groups. Demographic, radiological, hematological, and operative data were compared. Logistic regression was used to identify independent predictors of rupture.

Results

Cyst rupture was observed in 19 patients (51.4%). Preoperative C-reactive protein (CRP) levels and the presence of fissure connection were significantly higher in the ruptured group (p < 0.05). Multivariate logistic regression revealed that fissure relationship was an independent predictor of rupture (odds ratio {OR}: 5.150; 95% confidence interval {CI}: 0.957-27.719; p = 0.049). Cyst size and location were not significant predictors in the adjusted model. The model showed acceptable fit (Hosmer-Lemeshow p = 0.094) and moderate predictive power (Nagelkerke R² = 0.447).

Conclusion

Fissure involvement is a key anatomical risk factor for pulmonary hydatid cyst rupture and should be carefully assessed during preoperative imaging. The early identification of fissure-associated cysts may guide surgical planning, reduce complications, and improve patient outcomes.

## Introduction

Cystic echinococcosis, commonly referred to as hydatid disease, is a zoonotic infection caused by the larval stage of *Echinococcus granulosus*. The disease remains endemic in various regions, including the Middle East, Mediterranean countries, Africa, and parts of South America and Central Asia, posing a persistent public health burden in both pediatric and adult populations [1-3]. While hepatic involvement is more frequent in adults, the lungs represent the most commonly affected organ in children, owing to the increased elasticity of lung parenchyma that facilitates rapid cyst expansion [4,5].

Pulmonary hydatid cysts (PHCs) may remain asymptomatic for extended periods, yet their potential for severe complications such as rupture into the bronchial tree or pleural cavity necessitates prompt clinical attention [6,7]. Rupture is the most feared complication and may lead to hydropneumothorax, hemoptysis, bronchopleural fistula, or even anaphylactic reactions [8,9]. Numerous studies have emphasized the role of cyst size, location, and intracystic pressure as possible contributors to rupture, but definitive predictive factors remain controversial [3,10,11]. In particular, cysts located in the right middle lobe and lingula have been shown to carry a higher risk of rupture, likely due to anatomical constraints and localized intrathoracic pressure dynamics [2].

Surgical intervention remains the cornerstone of treatment, with lung-sparing techniques such as cystotomy and capitonnage being preferred, especially in pediatric patients [7,12,13]. However, the presence of ruptured cysts may complicate surgical procedures and increase postoperative morbidity rates, which underscores the importance of identifying predictors of rupture and refining preoperative risk stratification [14,15].

The aim of this study is to retrospectively evaluate the clinical, radiological, and perioperative features of patients operated on for pulmonary hydatid cysts in our center over a 10-year period and to determine the association between cyst rupture and preoperative parameters such as inflammatory markers, radiological findings, and anatomical localization.

## Materials and methods

Study design and population

**Figure 1:**
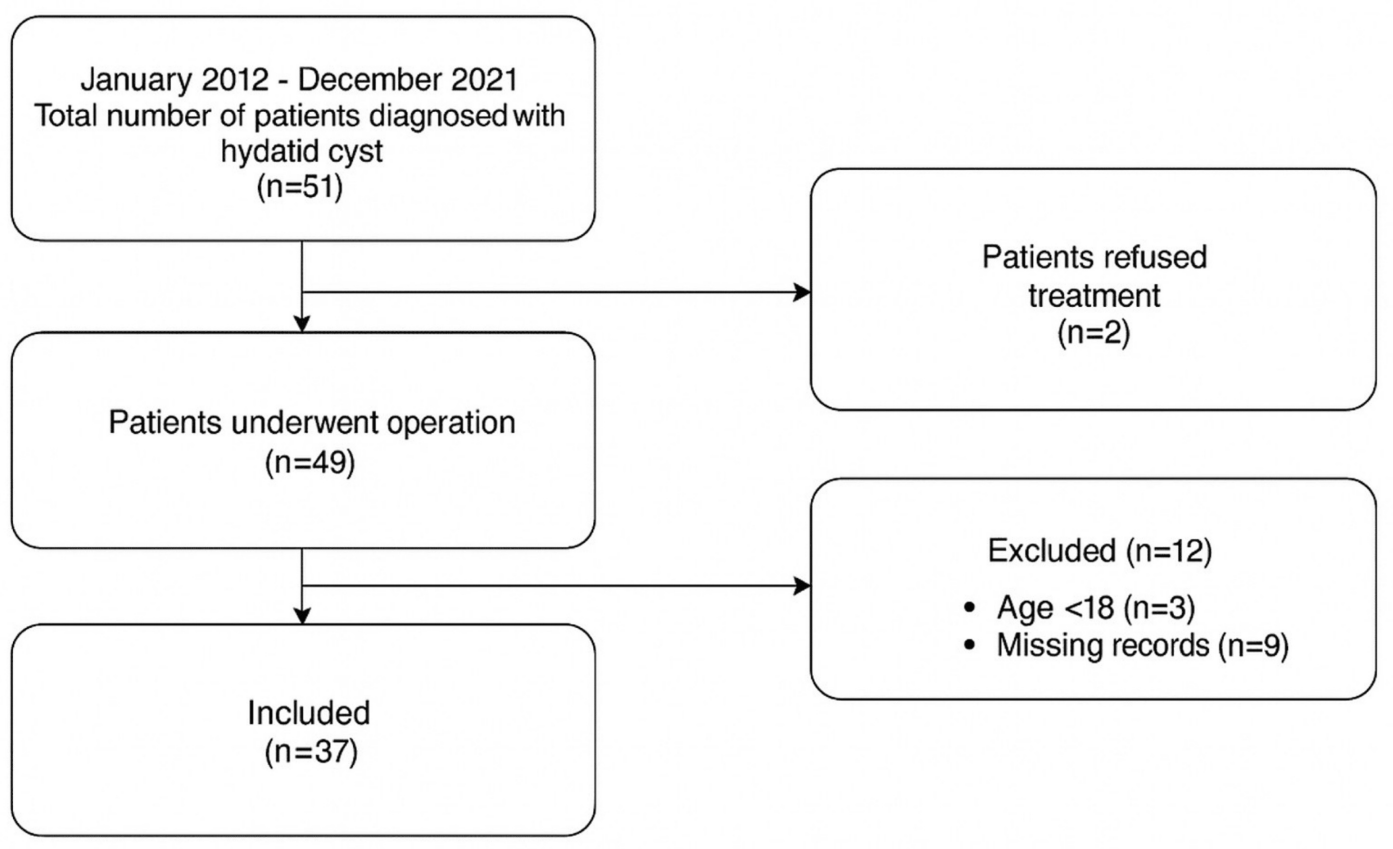
Flow chart of the study

This retrospective study included patients aged 18 years or older who underwent surgery for pulmonary hydatid cysts at our institution between January 2012 and December 2021. Only those with complete medical records and histopathological confirmation of hydatid disease were enrolled. Patients under the age of 18, those with incomplete medical records, or those without postoperative histopathological confirmation of hydatid disease were excluded from the study. The patient selection process is summarized in the flow diagram (Figure 1).

Ethics

The study was performed in accordance with the principles of the Declaration of Helsinki. Ethical approval was obtained from the local ethics committee (protocol code, 2022/8; committee number, 72867572-050.01.04-196247), and written informed consent was obtained from all the participants. The Suleyman Demirel University Faculty of Medicine Clinical Research Ethics Committee issued approval 196247/8.

Data collection

Preoperative data were extracted from electronic health records, including thoracic computed tomography (CT) scans, complete blood count (CBC), and C-reactive protein (CRP) levels. Hematological data focused on white blood cell counts, neutrophils, lymphocytes, eosinophils, neutrophil-to-lymphocyte (Ne/Ly) ratio, and CRP. Patients were divided into two groups based on the presence or absence of cyst rupture at the time of surgery.

CT images were obtained by a 128-slice multidetector CT (MDCT) (Somatom Definition AS, Siemens Healthcare, Erlangen, Germany) device. The following are the technical parameters of CT examination: Detector collimation is 128 × 0.6 mm, tube voltage is 120 kV, slice thickness is 1 mm, pitch value is 0.8, matrix is 512 × 512, and field of view (FOV) is 30-35 cm. The radiological characteristics of hydatid cysts were examined and recorded by a radiologist with 10 years of experience in thoracic radiology. Radiological parameters included cyst size, number of cysts, localization (lobe-specific), the presence of collapsed membrane, fissure or pleural involvement, and bilaterality.

Surgical procedures and classification

All surgeries were performed via thoracotomy. Surgical approaches consisted primarily of cystotomy with capitonnage, wedge resection, or lobectomy, based on intraoperative findings and the extent of parenchymal damage. The presence of rupture, fissure connection, and coexisting complications were documented perioperatively.

Statistical analysis

The distribution of continuous variables was assessed using the Kolmogorov-Smirnov test. Descriptive statistics were expressed as mean ± standard deviation for normally distributed variables, median (interquartile range) for non-normally distributed variables, and frequency with percentage for categorical data. For intergroup comparisons, the independent samples t-test was used for variables showing normal distribution, while the Mann-Whitney U test was applied for non-normally distributed variables (e.g., eosinophil count, neutrophils, lymphocytes, CRP, Ne/Ly, and age). Categorical variables were compared using the chi-square test or Fisher’s exact test, as appropriate. To identify independent predictors of cyst rupture, binary logistic regression analysis was performed. Variables that were significant in univariate analysis were included in the multivariate model. The goodness of fit of the model was evaluated using the Hosmer-Lemeshow test, and model performance was expressed as Nagelkerke R². A two-sided p-value of <0.05 was considered statistically significant. All analyses were conducted using IBM SPSS Statistics for Windows, version 27.0 (IBM Corp., Armonk, NY).

## Results

**Figure 2:**
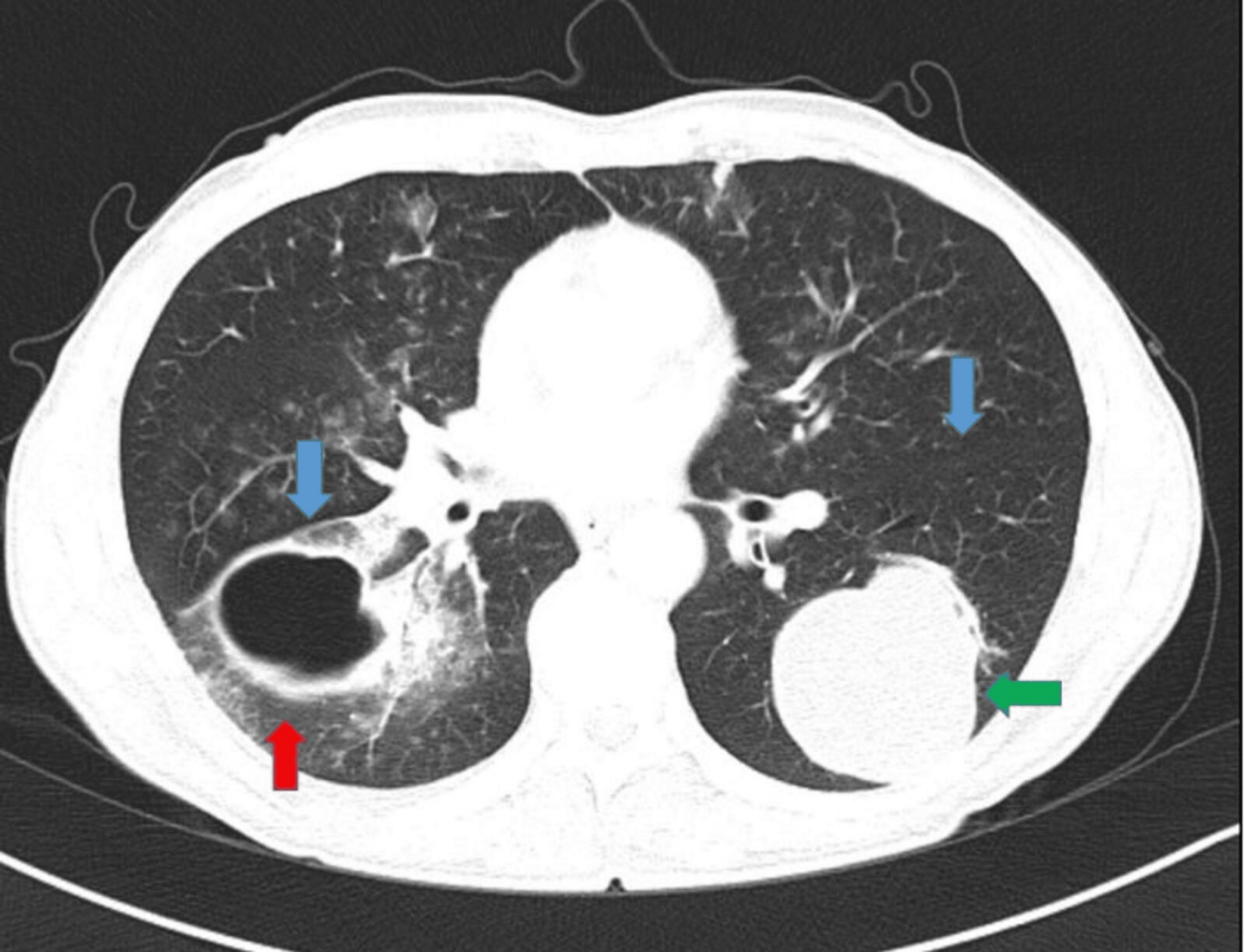
Thorax computed tomography parankim window shows bilateral hydatid cysts Major fissures are marked with blue arrows. The red arrow shows a perforated cyst related to a fissure. The green arrow shows an intact cysts not related to a fissure

A total of 37 patients (12 men and 25 women) were included in the study, with a mean age of 41.6 ± 15.4 years (range: 7-72). Patients were classified based on the presence of cyst rupture. Rupture was observed in 19 patients (51.4%). Radiological evaluation revealed that several ruptured cysts were located adjacent to interlobar fissures. Figure 2 illustrates a representative axial thoracic CT image showing a cyst in close proximity to the fissure, supporting the anatomical association between fissure involvement and rupture. Accompanying complications such as pleural effusion, pneumothorax, itching, and skin rash were present in 23 patients (62.2%). The most commonly performed surgical technique was capitonnage (86.5%). Right-sided cysts were seen in 17 patients, left-sided in 14 cases, and bilateral involvement in six cases. Detailed demographic and cyst characteristics are summarized in Table 1.

**Table 1:** Demographic and clinical characteristics of the patients SD: standard deviation

Variables		n (%)
Gender		
Female		25 (32.4)
Male		12 (67.6)
Age (Mean ± SD)	41.59 ± 15.37	
Cyst Characteristics		
Side		
Left		14 (37.8)
Right		17 (45.9)
Bilateral		6 (16.2)
Placement		
Central		3 (8.1)
Peripheral		29 (78.4)
Both		5 (13.5)
Fissure Relationship		
Yes		19 (51.4)
No		18 (48.6)
Size		
Small		14 (37.8)
Medium		17 (45.9)
Large		6 (16.2)
Pleura Relationship		
Yes		35 (94.6)
No		2 (5.4)
Perforation		
Yes		18 (48.6)
No		19 (51.4)
Complications		
Yes		23 (62.2)
No		14 (37.8)
Operation Technique		
Capitonnage		32 (86.5)
Wedge		4 (10.8)
Lobectomy		1 (2.7)
Localization		
Right Upper		2 (5.4)
Right Medium		5 (13.5)
Left Upper		6 (16.2)
Right Lower		7 (18.9)
Left Lower		4 (10.8)
Right Upper and Lower		2 (5.4)
Right Upper and Left Upper		1 (2.7)
Right Upper and Left Lower		1 (2.7)
Right Lower and Left Upper		1 (2.7)
Right Lower and Left Lower		1 (2.7)
Left Upper and Left Lower		4 (10.8)
3 and More (Multiple)		3 (8.1)

When parameters were compared according to rupture status, preoperative CRP levels were significantly higher in the ruptured cyst group (p < 0.05). Similarly, the presence of a fissure connection was significantly associated with rupture (p < 0.05). Among patients who had preoperative complications, 80% developed postoperative complications, indicating a statistically significant relationship (p < 0.05). No significant differences were found for other hematological or radiological parameters between the two groups (p > 0.05). Detailed results are presented in Table 2.

**Table 2:** Laboratory and clinical parameters according to rupture status ^β^Group comparisons were performed using the independent samples t-test. The t-values and corresponding p-values are presented ^π^Mann-Whitney U test was used for group comparisons; the test statistic (U) is reported ^£^Pearson’s chi-square test was applied for all comparisons (df = 1); χ² and p-values are reported ^µ^Fisher’s exact test CRP, C-reactive protein; Ne/Ly, neutrophil-to-lymphocyte; SD, standard deviation; df, degrees of freedom

	Overall (n = 37)	Perforation		Test Statistics	P-value
		Yes (n = 19)	No (n = 18)		
Laboratory (Mean ± SD)					
Eosinophil	0.57 ± 16	0.93 ± 1.27	0.20 ± 1.16	213.500^π^	0.199^π^
Neutrophil	6.53 ± 0.53	7.68 ± 3.90	5.31 ± 1.85	225.000^π^	0.105^π^
Lymphocytes	1.9 ± 0.99	1.86 ± 0.55	2.07 ± 0.65	137.000^π^	0.313^π^
Preoperative CRP	9.8 (1.1-242)	4.75 (1.1-146)	19.7 (3-242)	236.500^π^	0.046^π^
Ne/Ly	4.17 ± 0.67	5.03 ± 1.09	3.25 ± 0.75	-1.343^β^	0.189^β^
Bilaterality (n, %)	6 (16.2)	3 (50)	3 (50)	0.005^£^	1.000^µ^
Fissure Relationship (n, %)	18 (48.6)	13 (72.2)	5 (27.8)	6.112^£^	0.013^£^
Pleura Relationship (n, %)	35 (94.6)	19 (54.3)	16 (45.7)	2.232^£^	0.230^µ^
Accompanying Complications (n, %)	23 (62.1)	16 (80)	7 (20)	8.072^£^	0.007^£^
Postoperative Complications (n, %)	5 (13.5)	4 (80)	1 (20)	1.899^£^	0.340^µ^

In univariate logistic regression, fissure relationship, preoperative complications, and cyst size were found to be significantly associated with rupture. These variables were included in the multivariate model. In the final model, only the fissure relationship remained a significant independent predictor (odds ratio {OR}: 5.150; 95% confidence interval {CI}: 0.957-27.719; p = 0.049). The model demonstrated good fit (Hosmer-Lemeshow test: χ² = 9.392, degrees of freedom {df} = 5, and p = 0.094), with a Nagelkerke R² of 0.447. Regression outcomes are shown in Table 3.

**Table 3:** Logistic regression model predicting cyst rupture OR, odds ratio; CI, confidence interval

Variables		Univariable				Multivariable			
		β	OR	95% CI	p	β	OR	95% CI	p
Age		0.004	1.005	0.951-1.061	0.871	-	-	-	-
Gender (Male)		0.055	1.057	0.166-6.736	0.953	-	-	-	-
Fissure Relationship		1.729	5.633	1.370-23.167	0.017	1.639	5.150	0.957-27.719	0.049
Complications		2.126	8.381	1.770-39.692	0.046	1.874	6.512	0.952-44.552	0.056
Cyst Size	5-10 cm	1.792	6.000	1.261-28.547	0.024	1.138	3.120	0.452-21.555	0.249
	>10 cm	0.916	0.363	0.363-2.500	0.363	-0.698	0.498	0.041-6.098	0.585

## Discussion

This study evaluated clinical and laboratory predictors of cyst rupture in patients undergoing surgery for pulmonary hydatid cysts. Among all variables, the presence of a fissure relationship emerged as a significant independent predictor of rupture. Patients with fissure involvement were found to have more than fivefold increased odds of cyst rupture (OR: 5.150; 95% CI: 0.957-27.719), even after adjusting for other factors. This finding suggests that the anatomical proximity of the cyst to interlobar fissures may predispose to early membrane weakening and parenchymal disruption.

Previous studies have addressed various risk factors for rupture, including cyst size, peripheral location, and intracystic pressure. Pediatric series report a 15%-31% rate for giant pulmonary cysts [16]. Some authors suggest that the rupture rate increases with increased cyst diameter due to the thinner lung parenchyma surrounding it. However, some authors observed no correlation between cyst diameter and rupture. Contrarily, Burgos et al. indicated that cyst enlargement was a negative risk factor for rupture [17]. Akgul Ozmen and Onat found that the unruptured group had a significantly larger mean cyst diameter than the ruptured group [18]. Özdemir et al. highlighted that ruptured cysts are often located peripherally and that their growth may lead to structural instability, facilitating communication with adjacent bronchovascular or pleural structures [2]. Similarly, Onal and Demir reported significantly higher rupture rates in cysts located in the right middle lobe and lingula, possibly due to the confined anatomical space and dynamic compression from neighboring lobes [4]. In our study, cyst diameter did not increase or indicate signs of impending rupture. However, while cyst size and location were considered in our analysis, neither remained statistically significant in the multivariate model. The fissure relationship, on the other hand, maintained its significance, aligning with observations from Hamouri et al., who noted that multisegmental cyst involvement and fissure-adjacent localization increase the risk of rupture and surgical complexity [6].

The potential mechanism behind this association may be explained by the anatomical dynamics of fissures. Interlobar fissures are areas of reduced resistance and mobility, making cysts in these regions more susceptible to mechanical stress during respiratory cycles. Additionally, partial contact with multiple lobes may create variable pressure gradients, promoting endocyst separation and fissure erosion over time [3]. At the fistula site, the intracystic pressure is unopposed, which may lead to the herniation of the endocyst membrane, the disruption of its integrity, and eventual rupture. This proposed mechanism, supported by Ashour et al., suggests that mechanical forces combined with anatomical vulnerability contribute to spontaneous cyst rupture [19]. Our findings are in line with this hypothesis and further support the notion that fissure-adjacent cysts may be predisposed to rupture due to localized structural weakness.

From a surgical perspective, fissure-adjacent cysts are also associated with increased intraoperative risk. Our data demonstrated that these patients not only had higher rupture rates but also tended to have more frequent postoperative complications, although not statistically significant. This observation supports the findings of Ahmadinejad et al., who identified fissure involvement as a potential marker for increased postoperative morbidity [5].

Contrary to some earlier studies suggesting that larger cysts are more likely to rupture, our analysis did not find size alone to be an independent predictor [2,7]. While univariate analysis showed an association, the effect was attenuated in the multivariate model, possibly due to confounding from fissure proximity or coexisting inflammation.

Overall, the data underscore the importance of detailed preoperative imaging not only to determine size and location but also to assess for fissure proximity. The identification of fissure involvement should alert the surgical team to a higher likelihood of rupture and may warrant expedited intervention or a modified intraoperative strategy.

Limitations

This study has several limitations. First, its retrospective and single-center design introduces inherent selection and information bias. The relatively small sample size may also limit the generalizability of the findings to broader populations. Certain parameters, such as the fissure relationship or the severity of complications, may be subject to inter-observer variability due to the lack of standardized radiological or intraoperative definitions. Additionally, preoperative imaging assessments were not performed using a uniform protocol, which may influence the consistency of anatomical evaluations. Therefore, larger, prospective, and multicenter studies are needed to validate our results and establish more robust predictive models.

## Conclusions

This study demonstrates that anatomical fissure involvement is a significant and independent predictor of rupture in patients with pulmonary hydatid cysts. The presence of a fissure connection when identified preoperatively through imaging may help clinicians prioritize patients for earlier surgical intervention, potentially reducing intraoperative challenges and postoperative complications. While cyst size and laboratory parameters alone were insufficient to predict rupture, incorporating anatomical characteristics into preoperative evaluation may enhance clinical decision-making. The early recognition and tailored management of fissure-related cysts may ultimately improve patient outcomes and reduce healthcare burden. These findings provide a novel perspective for thoracic surgeons and radiologists in endemic regions, where the early identification of rupture-prone cysts can meaningfully influence treatment strategy. Incorporating fissure evaluation into routine preoperative imaging protocols may facilitate timely surgical decision-making and improve the safety of interventions. Additionally, the anatomical insight gained from this study supports a more individualized approach to patient care, moving beyond size-based risk models. Continued investigation with larger cohorts will be essential to validate these results and potentially integrate fissure-based risk assessment into clinical guidelines for hydatid disease. Pulmonary hydatid cysts still commonly occur in developing countries. In children, lung involvement is more common, and hydatid cyst growth is faster than in adults. Surgical treatment is the gold standard and depends on a number of factors, including the size of the cyst, rupture, solitary or multiple, unilateral or bilateral, and the degree of lung parenchymal destruction. Surgical management should include cystotomy and capitonnage with the preservation of the lung parenchyma as much as possible. Modified capitonnage technique for giant hydatid cysts is effective in reducing complication rates.
